# Graphene Nanoribbon Based Thermoelectrics: Controllable Self‐ Doping and Long‐Range Disorder

**DOI:** 10.1002/advs.201600467

**Published:** 2017-03-31

**Authors:** Huashan Li, Jeffrey C. Grossman

**Affiliations:** ^1^ Department of Materials Science and Engineering Massachusetts Institute of Technology 02139 Cambridge MA USA

**Keywords:** 2D‐material, density functional theory, graphene nanoribbons, helical architectures, thermoelectric

## Abstract

Control of both the regularity of a material ensemble and nanoscale architecture provides unique opportunities to develop novel thermoelectric applications based on 2D materials. As an example, the authors explore the electronic and thermal properties of functionalized graphene nanoribbons (GNRs) in the single‐sheet and helical architectures using multiscale simulations. The results suggest that appropriate functionalization enables precise tuning of the doping density in a planar donor/acceptor GNR ensemble without the need to introduce an explicit dopant, which is critical to the optimization of power factor. In addition, the self‐interaction between turns of a GNR may induce long‐range disorder along the helical axis, which suppresses the thermal contribution from phonons with long wavelengths, leading to anomalous length independent phonon thermal transport in the quasi‐1D system.

## 1 Introduction

2D materials such as graphene sheets,^1^, ^2^, ^3^ graphene nanoribbons (GNRs),^4^, ^5^, ^6^, ^7^ molybdenum dichalcogenides,^8^, ^9^ and phosphorene (monolayer black phosphorus)^10^ have been computationally proposed as promising building blocks for thermoelectric (TE) materials, although their realization in experiment remains a challenge due to the extrinsic scattering sources originating from material imperfections.^8^, ^11^ In addition to the modification of intrinsic quantum confinement and anisotropy of 2D materials,^10^ the control over architecture may serve as an attractive means to promote device performance,^1^ because the all‐surface character of 2D materials offers opportunities to strengthen interfacial effects in an organized ensemble, and the flexibility of 2D structures provides a large phase space for building tailored nano‐ and mesoscale architectures.

While still in its early stages, recently developed synthesis techniques reveal the possibility to realize complex 2D‐material architectures. With regard to ensemble regularity, both vertical^12^, ^13^, ^14^, ^15^, ^16^ and lateral^14^, ^17^, ^18^, ^19^, ^20^, ^21^, ^22^ heterojunctions based on 2D materials have been fabricated with atomic scale precision by chemical‐vapor deposition^12^, ^14^, ^21^ and dip‐pen nanolithography^22^ techniques. Examples of complex architectures of an individual material include helical twists which have been observed in graphene nanoribbons (GNRs) confined by carbon nanotubes (CNTs),^23^, ^24^ and more complicated 3D mesostructures produced by compressive buckling,^25^ self‐assembly,^26^ and polar‐surface‐driven growth^27^ approaches.

As an example of exploring material architecture, a recent theoretical study predicted that nanopatterning via chemical functionalization can enhance the optimized ZT of graphene significantly, due to the increased Seebeck coefficient and reduced thermal conductivity.^1^, ^3^ However, such potential relies on the ability to control precisely the doping level without the assistance of an external field. Yet, doping a 2D material is particularly difficult because if the interaction is weak as in the case of surface transfer doping, the doping level is limited by the steric repulsion between the absorbed dopant molecules and the system may not process long‐term stability; if the interaction is strong as in the cases of substitutional doping, the scattering of carriers will be severe and the defect density will be high.^28^, ^29^

In this work, we explore the impact of the material ensemble and architecture on TE properties, with emphasis on understanding the tunability of doping and thermal conductivity. Among the numerous derivatives of 2D materials, GNRs are chosen as our prototype systems because their electronic structure can be tailored by both geometry and passivation, which makes them especially attractive to provide desirable properties for electronic and spintronic applications.^30^, ^31^, ^32^ In addition, GNRs can form systems with various architectures despite the minimal complexity of the material itself.

**Figure 1:**
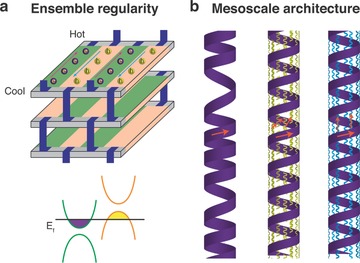
Schematics to illustrate examples of potentially emerging properties via material architecture design. a) Precise tuning of electronic structure in an ensemble of functionalized GNRs with a single‐sheet architecture, wherein a type‐III interface is formed between the donor (in orange) and acceptor (in green) GNRs. The top subpanel illustrates the proposed TE device, with the metal contacts displayed in dark blue areas and the current directions denoted by arrows. The bottom subpanel shows the energy level alignment, with the electron and hole doping ranges presented by the filled areas in purple and yellow, respectively. b) Modification of vibrational channels via self‐interactions in single GNRs with the helix architecture. The left subpanel presents the long‐range disorder in a GNR helix due to the aggregation of turns. The middle and right subpanels illustrate the additional phonon transport paths through the close but separate ligands and the ligands bridging the neighboring turns respectively. The red arrows denote the direction of the microscopic heat fluxes.

With multiscale simulations, we probe the electronic and thermal properties of different systems composed of functionalized GNRs in single‐sheet and helical architectures at room temperature (*T* = 300 K). Our results suggest that appropriate design of the GNRs leads to precise tuning of the electronic structure and modification of vibrational channels via self‐interactions. For example, with the single‐sheet architecture, type‐III electronic interfaces can be established to achieve controllable ground state doping while avoiding effects that can arise from an extrinsic dopant (**Figure** **1**a). With the helical architecture, thermal conductivity can be affected by long‐range disorder and additional transport paths (Figure 1b). While sophisticated control is required to achieve such premise in reality, our analysis of prototype TE active layers indicates that emergent phenomena dependent on the modification of material architecture may serve as a potential means to improve device properties.

## 2 Results and Discussion

**Figure 2:**
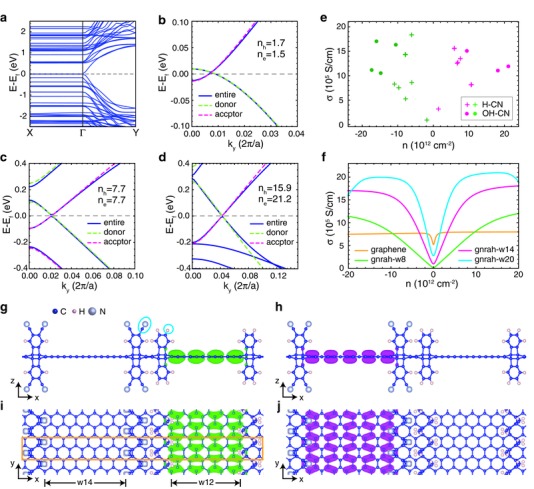
Modification of doping levels in GNR ensembles with the single‐sheet architecture. Here we use the notation “gnraβ‐wγ” to represent the GNR functionalized by benzene ligands with β endgroup, with the cross‐section of the conjugation area in the *x* direction containing γ C atoms. a) Bandstructures of the functionalized graphene sheet with gnraH‐w12 and gnraCN‐w14 as donor and acceptor, respectively. The *k*‐points shown are Γ = (0,0,0), *X* = (1/2,0,0), *Y* = (0,1/2,0). Comparisons between the bandstructures of the functionalized graphene sheets and their GNR components along the Γ–*Y* direction near the Γ point, corresponding to relatively b) low, c) medium, and d) high doping levels. The electron/hole doping levels *n*_e/h_ are in units of 10^12^ cm^−2^. e) Electrical conductivities and doping levels attained by tuning the GNR widths and the ligands. The doping concentration *n* is defined as the partial charge localized at each GNR divided by its area. The legend β1–β2 means the end groups of the ligands in the donor and acceptor GNRs are β1 and β2, respectively. f) Electrical conductivities of graphene and functionalized GNRs with various widths. g,h) Side and i,j) top views of the highest VB wavefunctions of gnraH‐w12/gnraCN‐w14 sheet at the *k*‐points (0.0,0.008,0.0) in green and (0.0,0.006,0.0) in magenta. The end groups of the ligands and the unit cell are highlighted by the blue ellipses and the orange rectangle.

The design of organized architectures using various types of building blocks has been shown by previous theoretical studies^1^, ^32^ to be a potential way to take advantage of the control over ensemble regularity due to the rich classes of phenomena incurred by interfaces. Following this strategy, we considered an ensemble of aligned GNRs chemically connected to each other (**Figure** **2**g–j), which effectively forms a functionalized graphene sheet with tunable energy level alignments between its 1D partitions. These particular benzene ligands are selected because they have been successfully added to the graphene surface by solution processing in recent experiments,^33^ and are arranged in the energetically favorable configurations suggested by previous calculations.^34^

In our prototype systems, a type‐III interface is established between the donor and acceptor GNRs, as shown by the crossing between the highest valence band (VB) of the donor and the lowest conduction band (CB) of the acceptor near the Γ point (Figure 2a–d). While the shapes of the bands (Figure 2b–d) and the wavefunctions (Figure 2g–j) of donor/acceptor GNRs are mainly preserved in the entire sheet as the ligands are only present near the edge of the GNRs, the absolute energy levels shift remarkably due to the potential alignment at the interface (Section B, Supporting Information), again indicating the importance of the ensemble regularity — precise control of the bandstructure requires precise control of the system architecture.

In order to tune the potential profile and attain a range of doping densities, two different groups of GNRs with various widths were used, with the end groups of their ligands switched between H, CN, and OH. Consistent with the previous study on graphene nanoroads,^1^ the electronic structures and transport properties of our isolated functionalized GNRs change significantly with the GNR widths, which could be classified into three categories (for gnraβ‐wγ, category‐I: mod(2γ,3)=0, category‐II: mod(2γ,3)=1, category‐III: mode(2γ,3)=2). When both the donor and acceptor GNRs are in category‐II with small bandgaps, the differences in chemical end groups of the ligands introduce sufficient potential drops inside the graphene sheets to form a type‐III energy level alignment and therefore incur ground‐state charge transfer. In these cases, the bandstructures of all samples are similar to each other, and the doping density decreases slightly with increasing domain width. When the donor and acceptor GNRs are in different categories, the doping density is sensitive to the band offset and spreads in a wide range. To gain extra tunability of the functionalized graphene sheets, the donor GNRs gnraH were replaced by gnraOH with electron donating chemical groups. As expected, a higher degree of charge transfer is reached, leading to relatively high doping densities (Figure S4, Supporting information). With such bandstructure engineering, a broad range of doping densities from 1.5 × 10^12^ to 2.1 × 10^13^ cm^−2^ becomes attainable (Figure 2e,f). While the extrinsic resources including charged impurities, neutral defects, interface roughness, graphene ripples, etc. serve as crucial scattering mechanisms for most devices based on 2D materials so far,^35^ they are likely to be suppressed by advanced synthesis techniques^36^ in the foreseeable future, which renders the room‐temperature carrier mobility limited by the intrinsic phonon scattering.^35^ In fact, an exceptional carrier mobility (10^4^ cm^2^ V^−1^ s^−1^) has been observed in graphene encapsulated by hexagonal boron nitride,^35^ approaching the value of 10^4^–10^5^ cm^2^ V^−1^ s^−1^ predicted by deformation potential theory assuming acoustic phonon is the dominant scattering mechanism.^35^ This method is applied in our work to probe the potential of ideal GNR systems. The results suggest that the electrical transport properties of a GNR depend on its category, width, and passivation, and are especially sensitive to the doping density (carrier concentration). For all of our prototype GNRs, the Seebeck coefficient peaks near zero carrier concentration owing to the large derivative of density of states (DOS), and then decays sharply with increasing doping density. In contrast, electrical conductivity increases with increasing width, with its optimal values achieved at relatively high doping densities. The power factor first increases then decreases with increasing doping density, and the peak positions are close to the ranges accessible by our proposed doping scheme. Modification of chemical end groups in ligands barely affects the electrical transport properties as the shapes of bandstructures are preserved even though the absolute energy levels may be shifted substantially (Section D, Supporting Information).

This tunability enables optimization of the electrical conductivity (σ), up to a value of 1.8 × 10^6^ S cm^−1^, more than twice that of the optimal σ in graphene. Only the conductivity in the *y*‐direction (along the GNR axis) was computed as carrier transport should be restricted to the 1D domain as revealed from the flat bandstructure in the *x*‐direction (perpendicular to the GNR axis, Figure 2a). Such a remarkably high conductivity can be attributed to the high DOS near the band edge, which substantially enhances the proportion of carriers with high velocity, even though the relaxation time is reduced simultaneously. We note that the deformation potential coupling constants of GNRs are assumed to be same as that of graphene following previous theoretical studies,^1^ and thus the calculated mobility can only be interpreted in a qualitative manner. In general, the attractive features of our prototype systems derive from the nature of self‐modulation doping—no explicit dopant and thus no extra scattering is introduced to the transport areas.

**Figure 3:**
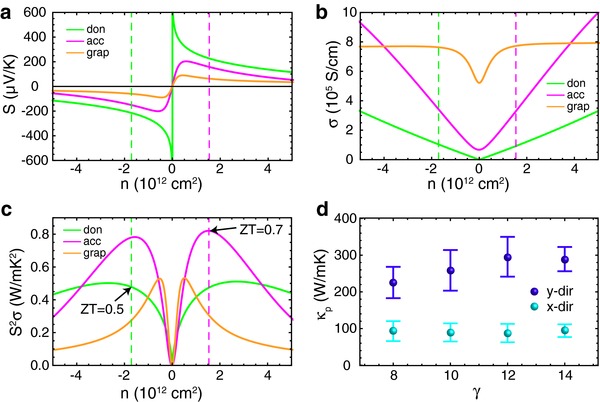
Properties of the prototype single‐sheet TE device. a) Seebeck coefficients, b) electrical conductivities, and c) power factors of graphene and the donor/acceptor GNRs of the sheet gnraH‐w12/gnraCN‐w14 at *T* = 300 K, with the doping levels denoted by the dashed lines. d) κ_p_ of functionalized graphene sheets composed of a single type of GNR obtained by the EMD method at *T* = 300 K. Each data point shows the average value of 10 samples, with the error bars illustrating the standard deviations.

Our doping scheme does not easily fit into either the electrical doping category with applied gate voltage or the chemical doping category with additional chemical species. In contrast to traditional chemical doping, the modification of functional groups in this study is used as a means to shift the local potential and thus stimulate internal charge transfer, rather than to introduce external carriers to the system. Further, our proposed patterning scheme enables the separation between the functional groups and carrier transport channels. While partially losing the flexibility of electrical doping because the doping density can no longer be tuned continuously and the doping density of donor and acceptor become correlated to each other, our doping scheme can be useful in circumstances where electrical doping is not feasible. Such a controllable, homogeneous and stable modulation doping scheme has the potential to address the intrinsic challenge of chemical doping in 2D materials and stimulate novel applications. In particular, the performance of a TE cell is especially sensitive to the doping level,^37^, ^38^, ^39^ which is a consequence of the need to optimize simultaneously the Seebeck coefficient (*S*) and electrical conductivity (**Figure** **3**a–c). As an example, we investigated a possible configuration of a TE cell composed of functionalized GNRs as shown in Figure 1a. Taking advantage of the ambipolar nature of graphene, current flows within the donor and the acceptor domains are in opposite directions when sharing the hot source and the cold drain, making it possible to connect the subcells in a serial manner (Figure 1a). Among the donor–acceptor pairs tested in this study (Section D, Supporting Information), the gnraH‐w12/gnraCN‐w14 sheet (Figure 2b,g–j) provides doping densities of 1.7 × 10^12^ and 1.5 × 10^12^ cm^−2^ for the donor and acceptor, respectively, which are sufficiently close to the optimal values (Figure 3c). Then, the phonon contribution to thermal conductivity (κ_p_) of the GNRs was calculated based on the fluctuation–dissipation theorem and rescaled to account for the long‐wavelength phonon contributions. The simulations were conducted on functionalized graphene sheets composed of a single type of GNR with the H end group, which accounts for the major impact of adjacent GNRs on κ_p_ while taking advantage of the efficient force field that is available only for C and H atoms. Consistent with the literature,^1^ the κ_p_ of GNRs are reduced by 5–6 times compared to that of pristine graphene (1810 W m^−1^ K^−1^)^1^ due to the boundary scattering and clamping effect (Figure 3d). We obtained ZT values of the donor (0.45) and the acceptor (0.75) at *T* = 300 K, with which the device ZT is estimated to be 0.55, much higher than the ZT of graphene (0.09) at its optimal doping level, mainly due to the reduction of κ_p_ (donor: κ_p_ = 295.7 W m^−1^ K^−1^, κ_e_ = 28.6 W m^−1^ K^−1^; acceptor: κ_p_ = 289.3 W m^−1^ K^−1^, κ_e_ = 40.5 W m^−1^ K^−1^). To further illustrate the importance of accurate control of the doping level, we computed the *S* ^2^σ and ZT at various doping levels for graphene and the functionalized GNRs (**Table** **1**). The results suggest that the deviation from the optimal doping level may suppress the device performance remarkably. In addition, the fluctuations in both *S* ^2^σ and ZT exhibit no correlation to their optimal values although a strong dependence on materials is observed, indicating that the tolerance of doping level should be considered along with the doping level itself for device optimization. In other words, a material with a high optimal figure of merit, which serves as the target in most computational design to date, is not necessarily optimal for device performance if the corresponding range of doping density is too narrow or too difficult to achieve in reality.

**Table 1:** *S* ^2^σ (in units of W m^−1^ K^−2^) and ZT at *T* = 300 K of n‐type graphene and H‐terminated functionalized planar GNRs with various doping levels (in units of 10^12^ cm^−2^). The optimal *S* ^2^σ and ZT values are obtained at optimal doping density (*n*)

	Opt. *n*	Opt. *S* ^2^σ	Opt. ZT	*S* ^2^σ				ZT			
*n*	–	–	–	0.2	1.0	5.0	10.0	0.2	1.0	5.0	10.0
Graphene	0.5	0.5	0.09	0.3	0.4	0.1	0.05	0.05	0.07	0.02	0.008
gnraH‐w8	2.0	0.6	0.7	0.2	0.5	0.4	0.1	0.2	0.6	0.5	0.1
gnraH‐w12	2.6	0.5	0.5	0.2	0.4	0.4	0.2	0.2	0.4	0.4	0.1
gnraH‐w14	1.5	0.8	0.8	0.2	0.8	0.2	0.009	0.1	0.7	0.2	0.007
gnraH‐w18	1.7	0.7	–	0.4	0.7	0.4	0.05	–	–	–	–
gnraH‐w20	1.2	0.8	–	0.06	0.8	0.06	0.0003	–	–	–	–

So far, we have examined the interactions between one material and another within an organized ensemble; it is also possible, though, to introduce self‐interactions within a single material in order to tune its novel properties by modifying the mesoscale architecture, which takes advantage of the different characteristic lengths of electron and phonon transport. A GNR helix is an appealing system to study the impact of mesoscale architecture, not only because of the fact that the absence of extra boundaries and abrupt geometric changes preserves the primary characteristics of the GNR, but also because the edge passivation provides opportunities for modifying the interactions between neighboring turns (Figure 1b).

**Figure 4:**
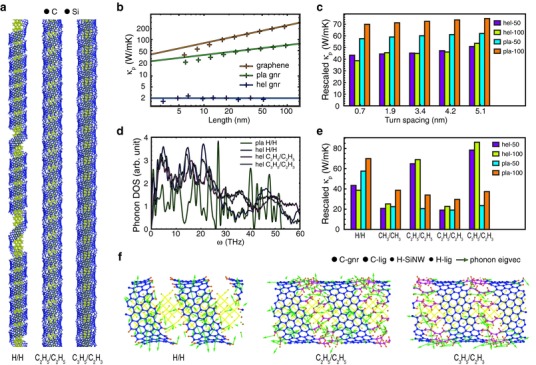
Thermal transport tuned by self‐interactions in helix GNRs. Here we use the notation “pla/hel β1/β2” to represent the planar/helix GNR with its two edges passivated by the ligands β1 and β2, respectively. a) Structures of GNR helices wrapping around frozen H‐SiNWs with lengths of 54 nm at the end of equilibration periods. b) Dependence of κ_p_ on the length along the transport direction for graphene, planar GNRs, and helix GNR(H/H)s. The fitting to the power law relation was carried out on the data points with *L* > 20 nm, because κ_p_ may be severely underestimated by the artificial boundary scattering in small systems. c) Comparisons between the rescaled κ_p_ of the helix GNR(H/H)s with various spacing between the turns and the κ_p_ of planar GNR(H/H)s with the same lengths. hel/pla‐50/100 means the length of the GNR is about 50/100 nm. d) Phonon DOS of planar and helix GNRs with their edges passivated by different ligands. e) Comparisons between the rescaled κ_p_ of the helix GNRs with various edge passivations and their planar GNR counterparts. f) Phonon modes of helix GNR(H/H), GNR(C_2_H_5_/C_2_H_5_), and GNR(C_3_H_5_/C_2_H_3_) with frequencies 15.7, 35.5, and 35.1 THz, respectively. The results in panels (a,b,c,e) were calculated by the reverse NEMD approach with frozen H‐SiNWs, while those in panels (d,f) were obtained by the EMD approach including the contributions from H‐SiNWs.

According to our simulations, isolated GNR helices are unstable without the presence of support scaffolds. The experimentally reported GNR helix‐CNT composites^23^, ^24^ exhibit strong coupling between the two materials, and thus are inappropriate for our purpose of investigating the influence of architecture alone on GNR materials. Instead, GNR helices wrapped around hydrogen‐passivated silicon nanowires (H‐SiNWs) were used as prototype systems (**Figure** **4**a). The width of the GNR is 10.3 Å, while the radius of the helix and the H‐SiNW was 9.3 and 6.7 Å, respectively. By considering a set of control systems of a planar GNR on top of a H–Si slab, we verified that the interactions originating from the H‐SiNW/GNR interface have a negligible effect on the electronic and thermal properties of the GNRs, and thus the H‐SiNW scaffold can be considered solely as a structural support, with no additional relevance to the emerging properties due to the change in architecture of the GNR from flat to helical (Section E, Supporting Information).

**Figure 5:**
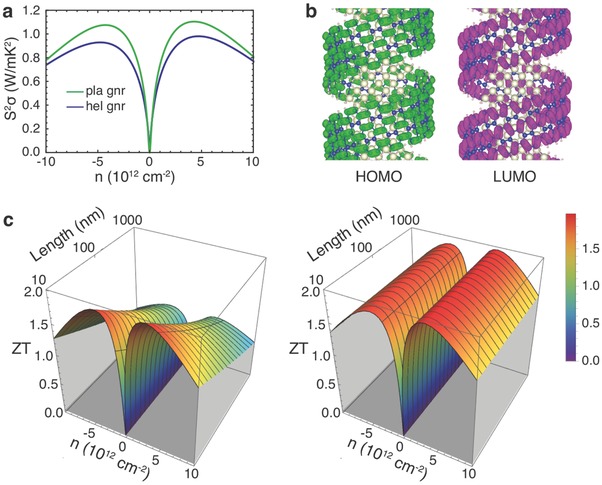
Properties of GNR(H/H) helices in thermoelectric applications. a) Comparison of the power factor at *T* = 300 K between the helix and the associated planar GNRs. b) VBM (in green) and CBM (in magenta) wavefunctions of the GNR(H/H) helix. c) Dependence of ZT on doping level and GNR lengths in planar and helix architectures.

Since the radius of a helical GNR is much larger than the sizes of the relevant atomic orbitals (≈2 Å), as perhaps expected the electronic properties of the helical GNR are similar to those of the corresponding planar GNR, with a slight drop in the computed conductivity due to the scattering induced by surface curvature (**Figure** **5**a,b and Section H, Supporting Information). By contrast, as revealed by the following discussions on thermal transport, the phonon contribution to the thermal conductivity (κ_p_) can either be suppressed by the scattering of long‐wavelength phonons which account for a large proportion of κ_p_, or enhanced by the additional transport paths through the side chain ligands in the helix architecture as illustrated by the cartoon in Figure 1b.

We note that there exists a long‐standing debate on whether the lattice thermal conductivity diverges with increasing system size in low‐dimensional materials.^40^, ^41^, ^42^, ^43^, ^44^, ^45^, ^46^ This has been clarified only recently by a theoretical study that exactly solves the Boltzmann transport equation for graphene,^42^ which is further verified by an empirical large‐scale nonequilibrium molecular dynamics (NEMD) simulation,^43^ and is consistent with the conclusion obtained by another theoretical study on CNT.^44^ Specifically, they found that κ_p_ increases substantially with increasing system size following a power or logarithmic law before reaching saturation in the thermodynamic limit with the size larger than a few mean free paths.^42^, ^43^, ^44^ The correct prediction of such behavior requires the consideration of both collective phonon excitations and three‐phonon scattering processes.^42^, ^44^ Since the mean free paths of the relevant systems are extremely large (hundreds of micrometers for graphene^42^ and tens of micrometers for CNTs^44^), it is prohibitive for our complicated helical structures. Therefore, rather than calculating the saturated κ_p_, we compared κ_p_ between the planar GNRs and the helix GNRs with the same lengths but different architectures, which should be sufficient to provide qualitative insights for the self‐interaction phenomena present in the helices.

With the GNR edges passivated by H atoms, the attraction between neighboring turns is strong due to the sufficient wavefunction overlap and the relatively weak steric repulsion between the ligands, which leads to aggregations of turns and thus a degree of disorder along the helix axis (Figure 4a). Such relatively long‐range disorder (at the length scale of tens of nm) serves as a source of strong scattering to the long‐wavelength phonons, resulting in an abnormal dependence of κ_p_ on the length (*L*) of the GNR—a nearly invariant κ_p_, in contrast to the power‐law relation for the planar GNR (Figure 4b). Excluding the pure geometric effect that a phonon needs to travel a longer distance compared to the direct transport via the axis direction in a helix structure, the rescaled κ_p_ (κ_p_/(sin θ)^2^) as defined in Section A in the Supporting Information) of the GNR helix with *L* = 54 nm is 40% lower than the κ_p_ of its planar counterpart (Figure 4c), and the reduction could be even more significant at larger *L* (Section F, Supporting Information). As expected, the reduction decreases with increasing spacing between turns due to the smaller curvature, while κ_p_ is still independent of *L* in most cases (Figure 4c).

In general, the reduction of κ_p_ associated with a helical architecture arises from two factors that distinguish it from the planar GNR. One is the variation of phonon frequency distribution—the population of low‐frequency optical modes has been substantially increased as shown in Figure 4d. This may result in resonance between optical and acoustic phonons that leads to stronger phonon–phonon scattering and shorter phonon lifetime. Such a mechanism is similar to the rattling effect, which has been demonstrated to be responsible for the short phonon mean free path and low κ_p_ in a variety of clathrates^47^ and skutterudites.^48^ The other factor responsible for the low κ_p_ can be interpreted by the scenario of phonon transport in the amorphous limit. Through systematic investigation of mixed crystals, Cahill et al. discovered that highly disordered solids exhibit similar lattice vibrations to those in amorphous solids, and therefore the heat transport of both materials can be appropriately described by Einstein's model assuming a random walk of thermal energy between neighboring atoms vibrating with random phases. They further pointed out that the presence of random, non‐central lattice distortion is efficient for suppressing κ_p_ toward the amorphous limit.^49^ Given the highly disordered aggregation patterns observed in our helical GNRs, the coherence between vibrations at relatively long distance is likely to vanish rapidly, which eliminates the contribution from long‐wavelength phonons and thus leads to length‐independent κ_p_.

When the H atoms at the two edges of a GNR helix are replaced by other carbon chains, the stronger steric effect reduces the degree of aggregation (Figure 4a), and the reduction of κ_p_ associated with the long‐range disorder may decrease accordingly, as seen in the cases of CH_3_/CH_3_ and C_2_H_5_/C_2_H_3_ wherein the interactions between the ligands are weak (Figure 4e and Section G, Supporting Information). By contrast, other ligands such as C_2_H_5_/C_2_H_5_ and C_3_H_5_/C_2_H_3_ may increase κ_p_ substantially (Figure 4e) by offering extra channels for phonon transport (Figure 4f), which is revealed by the new phonon DOS peaks in the range of 30–40 THz (Figure 4d). In particular, the C_2_H_5_/C_2_H_5_ ligands are flexible and sufficiently close to each other for efficient heat transport; while the C_3_H_5_/C_2_H_3_ ligands with conjugating C=C double bonds at the end of the chains tend to react with each other and bridge the neighboring turns.

Based on the above analysis, the design of the mesoscale architecture opens an opportunity to both promote and suppress the heat transport while roughly preserving the electronic properties, which could potentially be applied to meet the requirements for a high κ in next generation integrated circuits and 3D electronics as well as a low κ in thermoelectric energy conversion.^50^ In this work, we focus on exploring the potential of architecture design in thermoelectric applications, which mainly take advantage of the reduced thermal conductivity caused by long‐range scattering. As a prototype system, we considered the application of H passivated GNR helices in thermoelectric cells at *T* = 300 K. On the one hand, the power factor is reduced only slightly by the helical architecture, as expected from the small variations of both the Seebeck coefficient and electrical conductivity (Figure 5a). On the other hand, while the ZT of the planar GNR is reduced by threefold with its length increasing from *L* = 10 to 1000 nm, the ZT of the helical GNR remains unchanged with increasing system length. Therefore, although the ZT of the 10 nm helical GNR is similar to that of its planar counterpart, the improvement in the helix architecture is substantial at *L* = 1000 nm (Figure 5c).

## 3 Conclusion

In summary, the comparison of single‐sheet and helical architectures of functionalized GNRs sheds light on the potential benefits in TE applications from material architecture design: precise tuning of electronic structure and modification of vibrational channels via self‐interactions. Specifically, a type‐III energy level alignment can be established in the single‐sheet architecture to achieve controllable and homogeneous modulation ground state doping without deleterious scattering from explicit dopants, which is important for optimizing electrical conductivity and thermoelectric response. For the helix architecture, thermal conductivity can either be decreased by the long‐range disorder that suppresses the thermal contribution from long‐wavelength phonons, or increased by the additional transport paths through ligands that are close to or interconnected to each other. Although only the TE applications for GNRs with various architectures have been considered in our work, and the assumptions made in our model induce uncertainties in predictions, the ubiquitous underlying principles should be possible to be applied in other novel designs.

## 4 Experimental Section

A combination of electronic structure calculations and the Boltzmann transport approach was employed to predict the electronic transport properties at room temperature. Standard ab initio calculations within the framework of density functional theory (DFT), implemented in the Vienna Ab Initio Simulation package (v5.3),^51^ were employed to calculate the atomic structure, DOS, and bandstructure. Plane‐wave and projector‐augmented‐wave type pseudopotentials^52^ were applied with the PBE exchange‐correlation functional.^53^ Since the band structure of the entire sheet near the band edge is similar to that of its GNR components, the doping density was determined by integrating the DOS from the Fermi level to the band edge of the donor/acceptor GNRs, which is justified by the matching between doping densities of the entire sheet and its components. Transport coefficients at 300 K were obtained by semiclassical Boltzmann theory with an energy dependent relaxation time^1^ using the Boltzmann Transport Properties package (modified from v1.2.5).^54^ Since acoustic phonon scattering is the dominant scattering mechanism for carriers in GNRs,^35^ the electron relaxation time was calculated by deformation potential theory.^1^ The electron–electron scattering processes, though, must be accounted for in the heat transport calculations, thus the thermal relaxation time was estimated based on a previous analysis of the reduced Wiedemann–Franz ratio.^55^

The phonon contributions to the thermal conductivities (κ_p_) of the functionalized graphene sheets and the planar/helical GNRs were calculated using equilibrium molecular dynamics (EMD) and nonequilibrium MD (NEMD) simulations, respectively, as implemented in the Large‐scale Atomic/Molecular Massively Parallel Simulator package (v2014).^56^

All simulations were carried out at 300 K with a time step of 0.05 fs. For the functionalized graphene sheets, the EMD approach based on the fluctuation–dissipation theorem was chosen to reduce finite‐size effects. The Einstein relation was applied,^57^, ^58^ with the microscopic heat flux recorded for 2 × 10^7^ MD steps (1 ns) within the NVE ensemble after a 100 ps equilibration period within the NVT ensemble. All interactions were described by the adaptive intermolecular reactive empirical bond order (AIREBO) potential.^59^

For the helical GNRs, the EMD method is inappropriate because the direction of the microscopic heat flux is ill‐defined. In addition, the length dependence of κ_p_, which reveals interesting physics for this architecture, could not be obtained by the EMD approach. Therefore, the reverse NEMD approach with the Muller–Plathe algorithm^60^, ^61^ was used instead. After the equilibration period, momentum exchange was performed every 10 fs within the NVE ensemble, and up to 1 ns simulation time was used to achieve a steady state before recording the temperature profile and energy flux. H passivated Si nanowires (H‐SiNW) were inserted as cores to the helical GNRs in order to sustain the helical structures with little influence on their properties. The interactions within the GNRs and within the H‐SiNWs were described by the AIREBO^59^ and the Tersoff^62^ potentials, while the interactions between them were described by a Lennard–Jones potential with parameters documented in the Dreiding force field.^63^ Finally, the phonon spectra were computed by the Fix‐Phonon package,^64^ with the dynamical matrix constructed by observing the displacements of atoms during the MD simulation. Further details regarding the computational methods are provided in Section A in the supporting information.

We note that a number of approximations have been applied in this work in order to make the estimation of the thermoelectric figure of merit possible, which inevitably introduce uncertainties, for example, in the accuracy of the DFT energy levels, the phonon thermal conductivity based on finite‐size classical MD, and the absence of defects and boundary disorder. Thus, rather than interpreted in a quantitative way, this study is more appropriate to be considered as an attempt to qualitatively explore the relative potential benefits of architecture design in 2D materials for thermoelectric applications.

## Supporting information

Supplementary
