# Supplementary material for: Graphene Nanoribbon Based Thermoelectrics: Controllable Self‐ Doping and Long‐Range Disorder
Source: Adv Sci (Weinh). 2017 Mar 31;4(8):1600467. doi: 10.1002/advs.201600467 (PMC5566246; doi:10.1002/advs.201600467)
Supplement: Supplementary file 1 — Supplementary [file ADVS-4-na-s001.pdf]

Copyright WILEY-VCH Verlag GmbH & Co. KGaA, 69469 Weinheim, Germany, 2017.

ADVANCED SCIENCE Open Access

Supporting Information

for *Adv. Sci.,* DOI: 10.1002/advs.201600467

Graphene Nanoribbon Based Thermoelectrics: Controllable Self- Doping and Long-Range Disorder

*Huashan Li and Jeffrey C. Grossman**

Copyright WILEY-VCH Verlag GmbH & Co. KGaA, 69469 Weinheim, Germany, 2016.

Supporting Information

Supporting Information for: Graphene Nanoribbon based Thermoelectrics: Controllable Self-doping and Long-range Disorder

*Huashan Li and Jeffrey C. Grossman**

A. Methods

Electronic structure and doping density

Standard ab-initio calculations within the framework of density functional theory (DFT) were implemented to obtain the electronic structure of our prototype systems, using the Vienna Ab Initio Simulation Package (VASP, v5.3).[1] Plane-wave and projector-augmented-wave (PAW) type pseudopotentials[2] with kinetic-energy cutoffs of up to 500 eV were applied, with the PBE exchange-correlation functional[3] and a 15 Å vacuum in any non-periodic direction. The structures were relaxed until all forces were smaller than 0.03 eV/Å. The bandstructure and density of states (DOS) of the functionalized graphene sheets (GNR ensembles with the single-sheet architecture) were computed using a 3×45×1 Monkhorst-Pack k-point grid,[4] while those of the graphene nanoribbons (GNRs) were calculated with a much denser grid containing 201 points, because both doping density and electrical transport properties are sensitive to the electronic structure. For the GNR helixes, a mesh containing 31 k-points was used to achieve convergence.

Due to the weak hybridization between the donor and acceptor orbitals, the bandstructure of an entire graphene sheet near the band edges is similar to those of its GNR components, which enables the precise decomposition of DOS to the contributions from the donor and acceptor GNRs. Since the dispersion along the x direction is negligible (directions as defined in Figure 2), the electronic structure of the whole k space could be represented by that along the $\Gamma - Y$ line. By fitting the bandstructures of the donor/acceptor GNRs to that of the entire sheet, the relative energy levels of the GNRs with respect to the fermi level were determined. The doping densities were then obtained by integrating the DOS from the fermi level to the band edges of the donor/acceptor GNRs.

Electrical transport property

With the electronic structures predicted by DFT simulations, the transport coefficients were obtained by semi-classical Boltzmann theory with energy dependent relaxation time[5,6] using the BoltzTraP package (modified from v1.2.5).[7] For the functionalized graphene sheets, the hole/electron transport coefficients were calculated using their donor/acceptor GNR components, because the BoltzTraP package does not account for band crossing, and the carrier concentrations of the entire sheets do not reflect the local charge distributions.

The electrical conductivity (σ), the electric part of the thermal conductivity (κ_e), and the Seebeck coefficient (S) were computed by[5,7]

$$\sigma = e^2 \sum_n \int \frac{d\mathbf{k}}{4\pi^3} \left(-\frac{\partial f(\epsilon_{n\mathbf{k}})}{\partial \epsilon_{n\mathbf{k}}} \right) \tau^e(\epsilon_{n\mathbf{k}}) v_{n\mathbf{k}} v_{n\mathbf{k}} \tag{1}$$

$$\kappa_e = \frac{1}{T} \sum_n \int \frac{d\mathbf{k}}{4\pi^3} \left(-\frac{\partial f(\epsilon_{n\mathbf{k}})}{\partial \epsilon_{n\mathbf{k}}} \right) \tau^p(\epsilon_{n\mathbf{k}}) v_{n\mathbf{k}} v_{n\mathbf{k}} \left(\epsilon_{n\mathbf{k}} - \mu\right)^2 \tag{2}$$

$$S = \left(\sigma^{-1}\right) \frac{e}{T} \sum_n \int \frac{d\mathbf{k}}{4\pi^3} \left(-\frac{\partial f(\epsilon_{n\mathbf{k}})}{\partial \epsilon_{n\mathbf{k}}} \right) \tau^e(\epsilon_{n\mathbf{k}}) v_{n\mathbf{k}} v_{n\mathbf{k}} \left(\epsilon_{n\mathbf{k}} - \mu\right) \tag{3}$$

where e is the electronic charge, T is the temperature, μ is the chemical potential, $v_{n\mathbf{k}} = (1/\hbar) \bigtriangledown_k \epsilon_{n\mathbf{k}}$ is the group velocity in the nth band at $\mathbf{k}$, $\epsilon_{n\mathbf{k}}$ is the energy eigenvalue, $f(\epsilon_{n\mathbf{k}})$ is the Fermi-Dirac function, $\tau^e(\epsilon_{n\mathbf{k}})$ and $\tau^p(\epsilon_{n\mathbf{k}})$ are the electron and phonon relaxation time respectively. All simulations were performed at 300 K, including bands in the energy range of 0.4 Ry around the fermi level.

Since acoustic phonon scattering is the dominant scattering mechanism in GNRs,[8] the relaxation time for electron could be estimated by deformation potential theory,[5,6]

$$\frac{1}{\tau^e(\epsilon_{\mathbf{k}})} = \frac{2\pi k_B T D_{ac}^2}{\hbar A \rho_m v_{ph}^2} \sum_{\mathbf{k}'} \delta(\epsilon_{\mathbf{k}} - \epsilon_{\mathbf{k}'}) \tag{4}$$

where k_B is the Boltzmann constant, D_{ac} is the deformation potential coupling constant, ρ_m is the mass density, v_{ph} is the sound velocity, A is the area of the sample, and $\sum_{\mathbf{k}'} \delta(\epsilon_{\mathbf{k}} - \epsilon_{\mathbf{k}'})/A$

is the density of states. Since the relevant orbitals are localized in the conjugating area, the effective area A was modified accordingly. $v_{ph} = 2.1 \times 10^6\, cm/s$ and $D_{ac} = 16\, eV$ [9–11] of pristine graphene were applied to all samples.

For very clean electronic systems, the Wiedemann-Franz ratio could be reduced substantially because the e-e scattering event contributes to the thermal relaxation rate but not the charge relaxation rate. Thus, the phonon relaxation time is approximated as[12]

$$\tau^p = \frac{\tau^e}{1 + \tau^e/\tau_{th}^{ee}} \tag{5}$$

where τ_{th}^{ee} is the relaxation time of the thermal current due to e-e collisions. According to the previous work that estimates the τ_{th}^{ee} based on quasiparticle lifetime,[12] the value doesn't change too much in the carrier concentration range that is relevant to our system (10^{12}-10^{13} cm^{-2}), therefore we set $\tau/\tau_{th}^{ee} = 0.1$ ps in all simulations.

Phonon transport property

The phonon contribution to thermal conductivity (κ_p) of the functionalized graphene sheet and the helix GNRs were calculated by classical molecular dynamics (MD) simulations, as implemented in the LAMMPS package (v2014).[13] All simulations were carried out at 300 K with a time step of 0.05 fs.

For the functionalized graphene sheet, equilibrium molecular dynamics (EMD) based on fluctuation-dissipation theorem was chosen to reduce finite-size effects. κ_p of the donor and acceptor domains were estimated by those of the corresponding graphene sheets composed of single type of domains.

According to the Einstein relation,[14,15] κ_p was computed from the fluctuations of the heat current,

$$\kappa_{p\alpha} = \frac{1}{k_B V T^2} \lim_{t\to\infty} \frac{1}{2t} \left\langle \left[R_\alpha(t) - R_\alpha(0)\right]^2 \right\rangle \tag{6}$$

where T is the temperature, V is the system volume set to the product of the sheet area

times the interplanar distance (3.35 Å) of graphene. $\langle [R_\alpha(t) - R_\alpha(0)]^2 \rangle$ is the mean square displacement of the integrated microscopic heat flux along the α direction with

$$R_\alpha = \sum_i \epsilon_i r_{i\alpha} \tag{7}$$

where ϵ_i and $r_{i\alpha}$ are the energy and position of the ith atom. The microscopic heat flux were recorded for 2×10^7 MD steps (1 ns) after a 100 ps equilibration period to reach convergence. 10 separate simulations with different initial conditions were averaged for each system. Since the difference between the κ_p calculated using a 2×6 supercell (about 50×25 Å) and those with larger supercells are less than 5% in our test case, the 2×6 supercell is adopted in all other samples.

Even though the second-generation reactive empirical bond order (REBO) potential[16] have been proved to be sufficient for graphene systems,[5] the more expensive adaptive intermolecular reactive empirical bond order (AIREBO) potential[17] is necessary for our cases, which appropriately describes the interactions between conjugating ligands. In order to account for the long-wavelength phonon contributions, the calculated κ_p were rescaled using graphene as a reference to the previous theoretical work that accounts for the long-wavelength phonon contribution by the Klemens Model.[5]

For the GNR helixes, reverse nonequilibrium molecular dynamics (RNEMD) simulations were performed with the Muller-Plathe algorithm which imposes the heat flux to form temperature gradient.[18,19] Since the GNR helixes by themselves are not stable in our MD simulations under an NVT ensemble, H passivated Si nanowires (H-SiNW) were inserted as cores to sustain the helix structures. The diameters of the H-SiNWs were chosen to be around 6 Å less than those of the GNR helixes to minimize the stress imposed by the H-SiNWs on the helixes, which is confirmed by the negligible change of relaxed helix diameter after insertion of H-SiNWs.

To study the length dependence of κ_p, various simulation boxes with periodic boundary

condition were used, which lengths along the heat transfer direction were set to twice of the corresponding GNR helixes. Each simulation box is divided into 50 slabs along the heat transfer direction. After a 100 ps equilibration period, momentum exchange between the coldest and the hottest slabs was performed every 10 fs. Our convergence tests confirm that this exchange frequency is sufficiently low such that κ_p is independent of the exchange frequency. The nonequilibrium steady states can be achieved within 1 ns simulation time for the longest GNR helix. The heat flux was then computed from the exchanged kinetic energy between two selected layers,

$$J = \frac{\sum_{N_{swap}} (m_h v_h^2 - m_c v_c^2)/2.0}{t_{swap}} \tag{8}$$

where $m_{h/c}$ and $v_{h/c}$ are the atomic masses and the velocities of the hot and cold atoms respectively, t_{swap} is the entire swap time, N_{swap} is the number of swaps. The temperature profile was calculated as

$$T_i = \frac{2}{3Nk_B} \sum_j \frac{p_j^2}{2m} \tag{9}$$

where T_i is the temperature of the ith slab, N is the number of atoms inside the slab, and p_j is the momentum of the jth atom. Finally, κ_p was obtained by

$$\kappa_p = \frac{J}{2A\partial T/\partial z} \tag{10}$$

where A is the cross section of heat transfer. For the combined helix and H-SiNW systems without constraint, $A = \pi(r+s/2)^2$, where r is the radius of the helix and s is the interplanar distance (3.35 Å) of graphene. For the combined helix and H-SiNW systems with frozen H-SiNW cores, $A = s \times w_p/sin\theta$, where w_p is the width of the associated planar GNR, and $\theta = arctan(\frac{lc}{2\pi r})$ with lc the distance between neighboring turns. The rescaled κ_p of a helix is defined as $\kappa_p/(sin\theta)^2$, which is just a convenient way of excluding the bare geometric factor for direct comparison with the associated planar GNR. The time intervals for calculating

κ_p were set to be sufficiently long such that both the heat flux and temperature profile are independent of the time interval.

We note that while the NEMD approach simulates the temperature gradient directly and introduces various degree of boundary scattering by modification of simulation box size, it does not account for the contributions from long-wavelength phonons. Nevertheless, NEMD simulation has been successfully applied to illustrate the power law dependence of thermal conductivity on system length in the range of about 10^2-10^3 nm[20,21] and the transition between ballistic and diffusive transport regioms[20,22] in quasi-1D materials including CNTs[20] and SiNWs,[21] which are consistent with both the predictions from theoretical analysis[23–27] and the violation of Fouriers' Law observed in experiments.[28,29] This indicates that the scattering of long-wavelength phonon transport may be similar to that of short-wavelength phonons, and that the NEMD method may capture the essence of the length dependent scattering phenomena, which enables us to qualitatively investigate the effects of self-interactions in GNR helixes.

Finally, the phonon spectra were computed by the Fix-Phonon package,[30] with the dynamical matrix constructed by observing the displacements of atoms during MD simulations. Convergence is reached with a simulation period of 400 ps and a length of about 330 Å.

Figure of merit

The power factor was calculated by

$$P = \sigma S^2 \tag{11}$$

where σ is the electrical conductivity, and S is the Seebeck coefficient. The figure of merit ZT of each material was obtained by

$$ZT = \frac{\sigma S^2 T}{\kappa_p + \kappa_e} = \frac{PT}{\kappa_p + \kappa_e} \tag{12}$$

where κ_p and κ_e are the phonon and electron contribution to the thermal conductivity respectively. Given the parameters from both donor and acceptor materials, the figure of merit of the entire system was evaluated as

$$ZT = \frac{(S_d - S_a)^2 T}{((\kappa_d/\sigma_d)^{1/2} + (\kappa_a/\sigma_a)^{1/2})^2} \tag{13}$$

B. Comparison of electronic structures between an entire graphene sheet and its GNR components

As illustrated by the prototype system gnraH-w8/gnraCN-w8 (notations as explained in Figure 2), while the shape of the bandstructure of the entire graphene sheet is similar to those of its donor/acceptor GNR components due to the weak orbital hybridization, the absolute energy levels of the GNRs are shifted significantly when embedded in the single graphene sheet (Figure S1), indicating that appropriate architecture is necessary to control the electronic properties of devices.

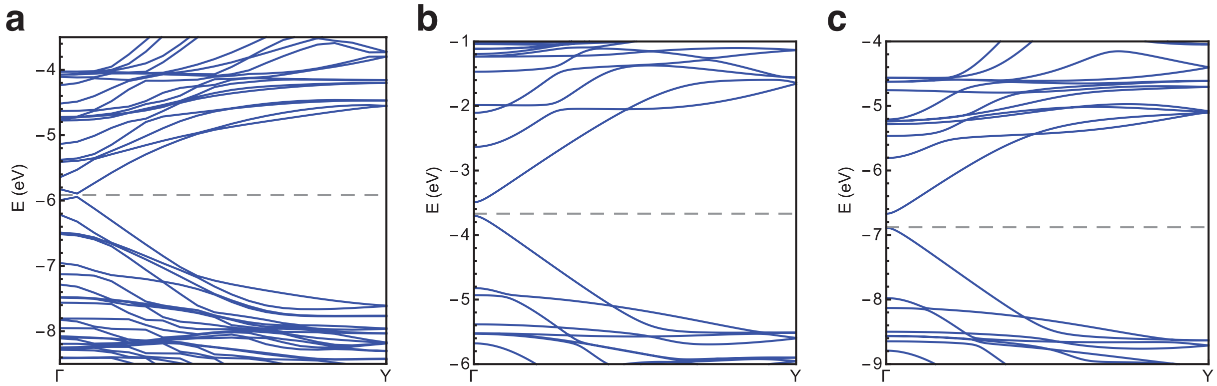

Figure S1: Bandstructures of (a) the entire graphene sheet gnraH-w8/gnraCN-w8, isolated (b) donor GNR gnraH-w8 and (c) acceptor GNR gnraCN-w8. The fermi levels are denoted by the dashed lines.

Given the similarity of the near-edge band structures and the wavefunctions between the entire graphene sheet and its GNR components (Figure 2, Figure S3), it is reasonable to de-

compose the charge of the entire graphene sheet to the contributions from the donor/acceptor GNRs, which allows us to determine the degree of doping caused by ground state charge transfer inside the single sheet. This hypothesis is further justified by the matching between the integrated DOS of the entire sheet and the sum over those of the corresponding isolated GNRs as shown in Figure S2.

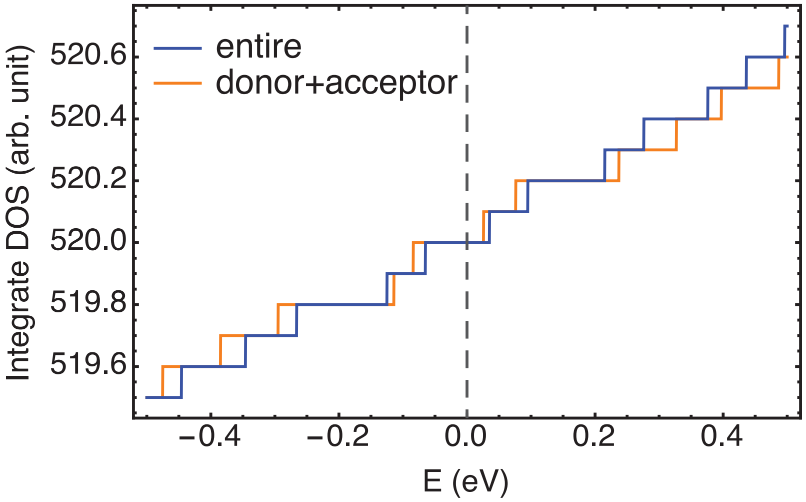

Figure S2: Integrated DOS of the entire sheet gnraH-w8/gnraCN-w8 and the sum over those of its donor/acceptor GNR components.

Consistent with the previous study on graphene nanoroads,[5] the electronic structures and transport properties of our functionalized GNRs change significantly with the GNR widths, which could be classified into three categories. In each category, the bandgap decreases with increasing width due to the weaker quantum confinement effects. In order to attain a range of doping densities, two different groups of GNRs with various widths were used, with the end groups of their ligands switched between H, CN and OH. As summarized in Table S1, while the band gap of a GNR mainly depends on its width, modification of the chemical end groups of the ligands may have substantial impacts on the absolute energy levels, which opens an opportunity to tune the energy level alignment via ligand exchange reactions in solutions.

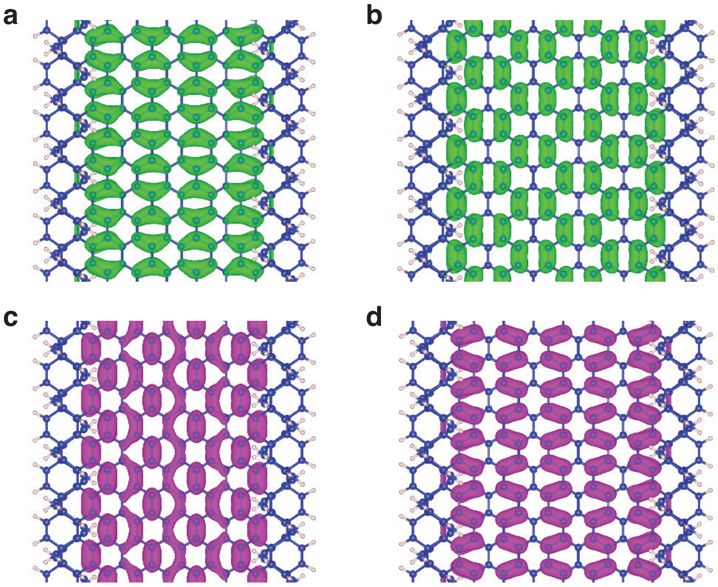

Figure S3: (a)(c)HOMO and (b)(d) LUMO wavefunctions of isolated GNRs (a)(c) gnraH-w12 and (b)(d) gnraH-w14.

C. Doping densities of functionalized graphene sheets with various passivations and domain widths

In order to illustrate the possibility to control the doping density of functionalized graphene sheets, we considered a series of prototype systems with various ligands and domain widths, and the electronic structures of the ones exhibiting ground state charge transfer characteristics are summarized in Figure S4 and Figure S5.

The impact of domain width was studied using the gnraH/gnraCN systems. When both the donor and acceptor GNRs are in category-I (gnraX-w6/w12/w18) with comparatively large bandgaps, the energy level alignments tend to be type-I or type-II, and thus the doping densities are zero. When both the donor and acceptor GNRs are in category-II (gnraX-w8/w14/w20) with comparatively small bandgaps, the differences in chemical end groups of the ligands introduce sufficient potential drops inside the graphene sheets to form a type-III energy level alignment and therefore incur ground-state charge transfer (Figure S4, 1st and 2nd rows). In these cases, the bandstructures of all samples are similar to each other, and

Table S1: HOMO, LUMO and band gap of functionalized GNRs (gnraβ-wγ) with various widths and ligands (vacuum level is zero). GNRs with $\beta = 6, 12, 18$ and those with $\beta = 8, 14, 20$ are in two different categories.

	HOMO (eV)	LUMO (eV)	gap (eV)
gnraH-w6	-4.09	-3.09	1.00
gnraH-w8	-3.70	-3.49	0.21
gnraH-w12	-3.99	-3.42	0.57
gnraH-w14	-3.79	-3.66	0.14
gnraH-w18	-4.00	-3.58	0.42
gnraH-w20	-3.86	-3.76	0.10
gnraCN-w8	-6.89	-6.67	0.22
gnraCN-w14	-6.40	-6.25	0.15
gnraCN-w20	-6.05	-5.91	0.14
gnraOH-w8	-4.11	-3.93	0.18
gnraOH-w14	-4.12	-4.00	0.11

the doping density decreases slightly with increasing domain width. When the donor and acceptor GNRs are in different categories, the doping density is sensitive to the band offset and spreads in a wide range (Figure S4, 3rd rows). To gain extra tunability of the functionalized graphene sheets, the donor GNRs gnraH were replaced by gnraOH with electron donating chemical groups. As expected, a higher degree of charge transfer is reached, leading to relatively high doping densities (Figure S5).

D. Electrical transport properties of GNRs with various widths and passivations

Because of the various quantum confinement, graphene and GNRs exhibit different electronic structures and electron relaxation time as shown in Figure S6, which result in distinct electrical transport properties (Figure S7). Note that our calculated density of states and relaxation time for graphene is consistent with previous simulations.[5]

The electrical transport properties of a GNR depend on its category, width, and passivation, and are especially sensitive to the doping density (carrier concentration). The Seebeck

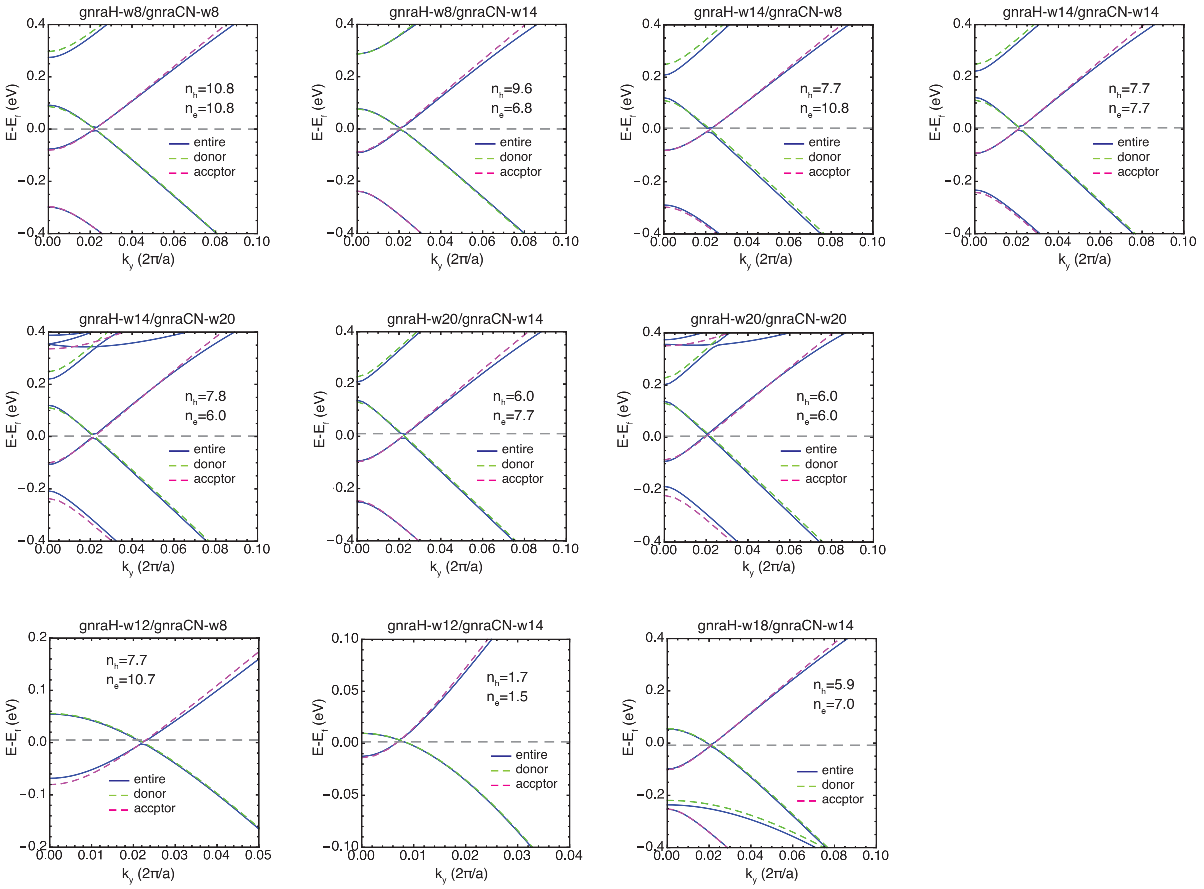

Figure S4: Bandstructures and doping density (in unit of 10^{12}cm^{-2}) of gnraH/gnraCN graphene sheets with various domain widths.

coefficient (S), electrical conductivity (σ), electron contribution to thermal conductivity (κ_e) and power factor ($S^2\sigma$) of our prototype GNR systems in the doping density ranges that could be achieved in our proposed single sheet architecture (Figure 2) are summarized in Figure S7 and Figure S8, and the following discussions focus on these relevant carrier concentration ranges.

For all of our prototype GNRs, the seebeck coefficient peaks near zero carrier concentration owing to the large derivative of DOS, and then decays sharply with increasing doping density. The S of GNRs in category-I is larger than that in category-II, while both decrease with increasing width (Figure S7 (a)(b)). In contrast, electrical conductivity increases with increasing width, with its optimal values achieved at relatively high doping densities. Notice

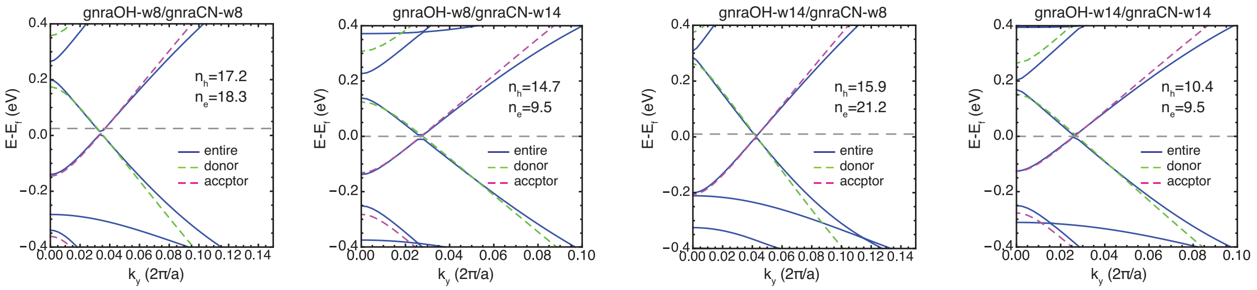

Figure S5: Bandstructures and doping density (in unit of 10^{12}cm^{-2}) of gnraOH/gnraCN graphene sheets with various domain widths.

that σ has a flatter profile near the optimal value in category-II GNRs than that in category-I GNRs, which may favor the applications that require high stability of σ (Figure S7 (c)(d)). The electron contribution to thermal conductivity has a comparably weak dependence on GNR width, and increases with increasing doping density (Figure S7 (e)(f)). While κ_e is generally considered to be much smaller than the phonon contribution in realistic system due to defect scattering, our results suggest that for extremely clean GNRs with acoustic phonon scattering serving as the dominant scattering mechanism, κ_e could be as large as 25% of the total thermal conductivity and thus should not be neglected. The power factor first increases then decreases with increasing doping density, and the peak positions are close to the ranges accessible by our proposed doping scheme (Figure S7 (g)(h)). The width of the peak and thus the stability decreases with increasing GNR width, and eventually becomes similar to that of pristine graphene. Modifications of chemical end groups in ligands barely affect the electrical transport properties as shown in Figure S8. That is because the shapes of bandstructures are preserved even though the absolute energy levels may be shifted substantially.

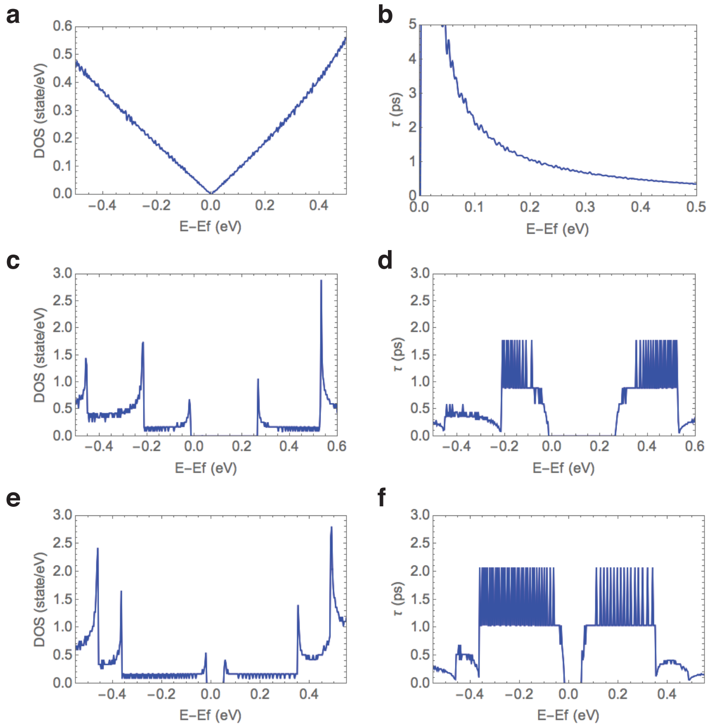

Figure S6: Density of states (a,c,e) and relaxation time calculated by deformation potential theory (b,d,f) for (a,b) graphene, (c,d) donor gnraH-w12, and (e,f) acceptor gnraCN-w14.

E. Comparison of phonon thermal transport between combined GNR-SiNW system and its components

In order to confirm that the thermal conductivities calculated by our MD simulations reflect the intrinsic properties of GNRs rather than the influences of GNR-SiNW interfaces, we compared the κ_p of combined GNR-SiNW systems and their components in both helix and planar architectures. As shown in Figure S9, with the helix architecture, the κ_p of both isolated SiNW and combined helix-SiNW system without constraint increase with increasing GNR length following the power law relation, while κ_p of the GNR helix wrapping around the frozen SiNW is almost independent to the GNR length. (here the cross sections of the SiNW and the GNR helix are set to that of the combine system for direct comparison.) Therefore, while the contribution from the GNR helix is more important in the short-length limit, κ_p of the combined system is mainly attributed to the transport in SiNW at relatively

long lengths.

By contrast, in the planar architecture where the planar GNRs lying on top of Si slabs, κ_p of the combined systems are similar to those of the associated isolated GNRs, which increases with increasing GNR length following the power law relation (Figure S10, the cross sections of the combine systems are set to those of the corresponding planar GNRs for direct comparison). That means the scattering induced by GNR-Si interfaces is weak, and should not be the main reason for the abnormal thermal transport properties observed in GNR helixes.

To further explore the influence of architecture on heat transport, we compared the phonon density of states (DOS) of the isolated planar GNR to that of the combined GNR helix-SiNW system (Figure S11). While the peaks of the GNR helix-SiNW system is generally broader than that of the planar GNR due to its larger geometric inhomogeneity, the peak positions are quite similar in both cases, implying that the scattering of the phonon modes rather than the mode distribution is responsible for the distinct thermal transport behaviors in different architectures.

Section F. Length dependent thermal conductivity of GNR helix

While the additional phonon scattering mechanism caused by the long range disorder affects the thermal conductivity only slightly for short GNRs, the reduction of κ_p in helix compared to planar architecture becomes non-negligible for GNRs longer than 100 nm as illustrated in Figure S12. Here the κ_p of our prototype GNR is estimated by extrapolation from the fitting to power law relation using the MD simulation data for short GNRs as shown in Figure 4(b). We assume the power law relation is still valid for GNRs with lengths up to 5000 nm at 300 K, based on a previous analytical study on CNT systems.[25]

Section G. Phonon DOS of helix-SiNW systems with various side chains

For obtaining strong self-interaction effects by tuning the GNR edge passivations, the ligands need to be chosen appropriately such that they are either chemically bonded or sufficiently close to each other as shown in Figure 4(f). Otherwise, the influence of the ligands would be weak because no new phonon transport channel is generated at the ligand-ligand interfaces (Figure S13).

Section H. Comparison of electronic properties between planar and helical GNRs

As shown in Figure S14, the surface curvature of the helical GNR weakly affects the electronic structure, leading to a slight decrease of both the electrical conductivity and electron contribution to thermal conductivity, while a slight increase of the Seebeck coefficient compared to the associated planar GNR.

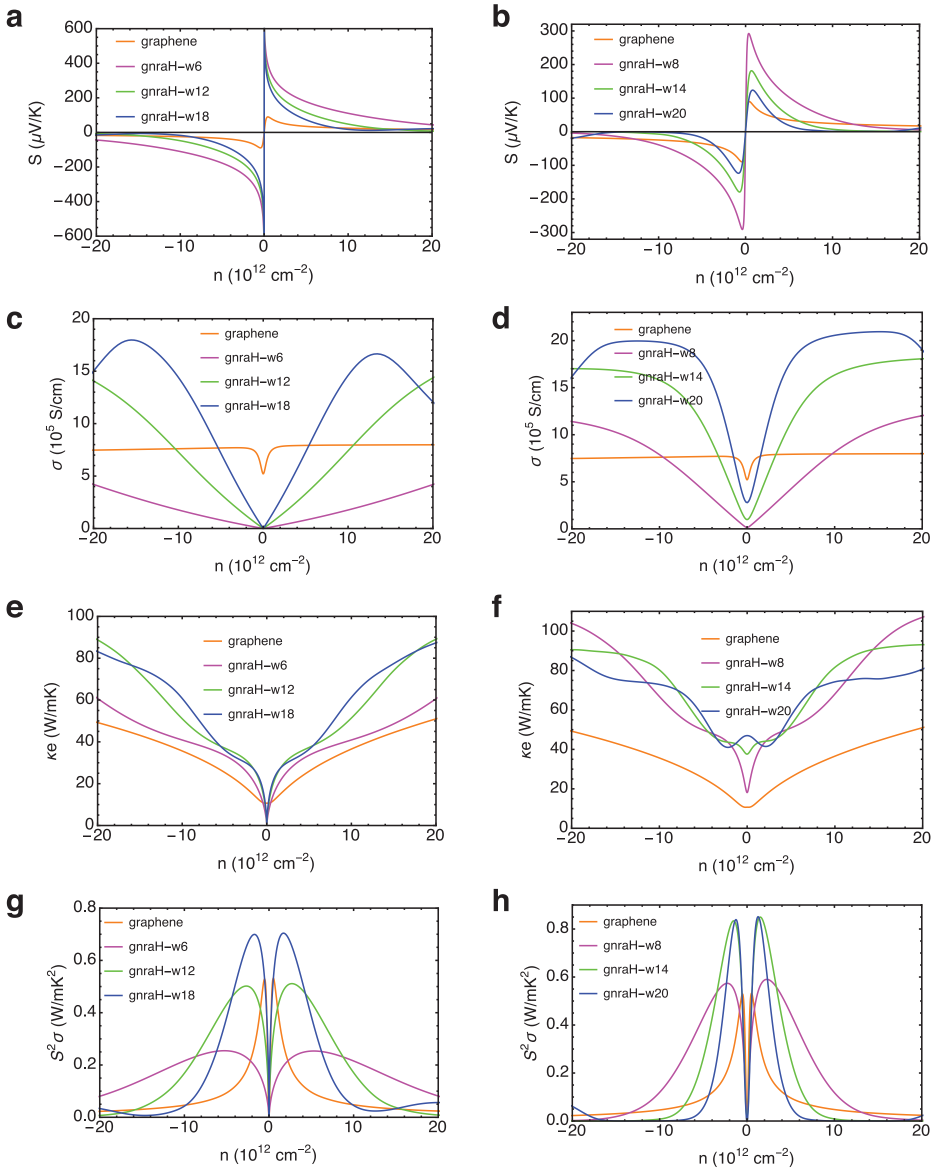

Figure S7: (a)(b) Seebeck coefficient (S), (c)(d) electrical conductivity (σ), (e)(f) electron contribution to thermal conductivity (κ_e) and (g)(h) power factor ($S^2\sigma$) with respect to carrier concentration in graphene, (a)(c)(e)(g) category-I, and (b)(d)(f)(h) category-II GNRs (gnraH) with various widths.

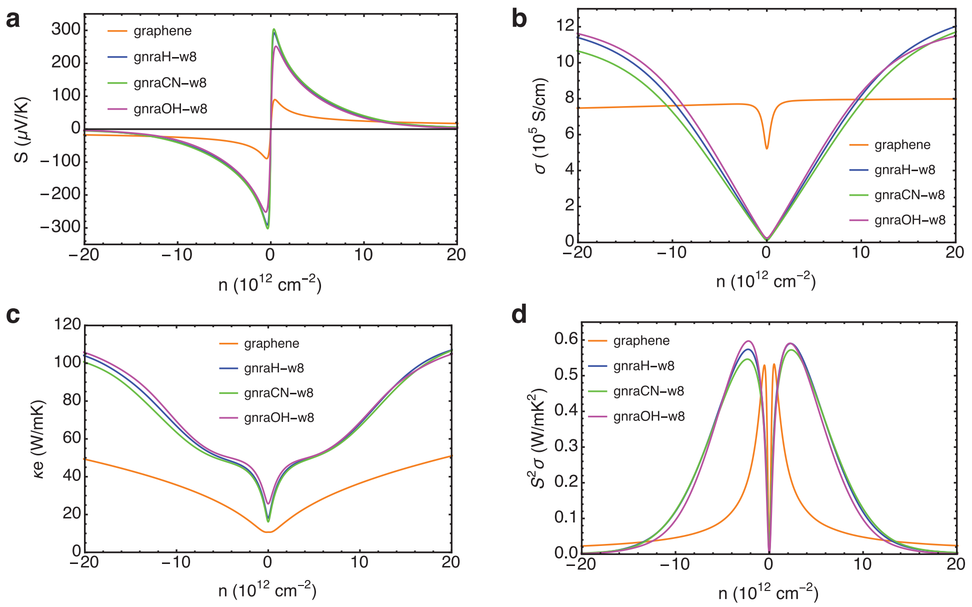

Figure S8: (a) Seebeck coefficient (S), (b) electrical conductivity (σ), (c) electron contribution to thermal conductivity (κ_e) and (d) power factor ($S^2\sigma$) with respect to carrier concentration in graphene and GNRs (gnraX-w8) with various ligands.

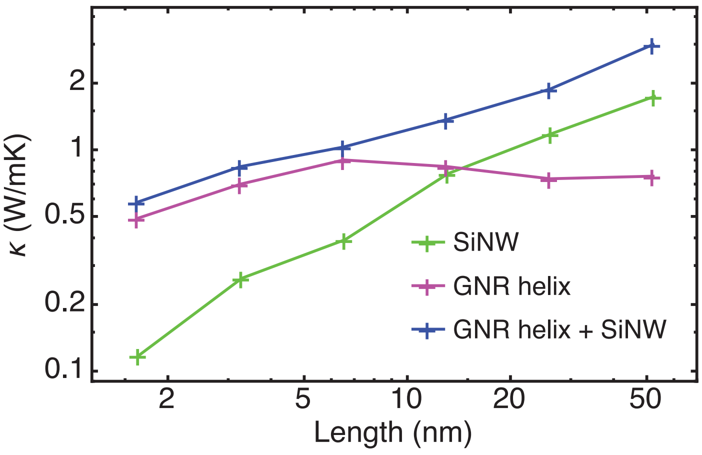

Figure S9: Length dependent κ_p of isolated SiNW, combined GNR helix-SiNW system with no constraint and with SiNW frozen.

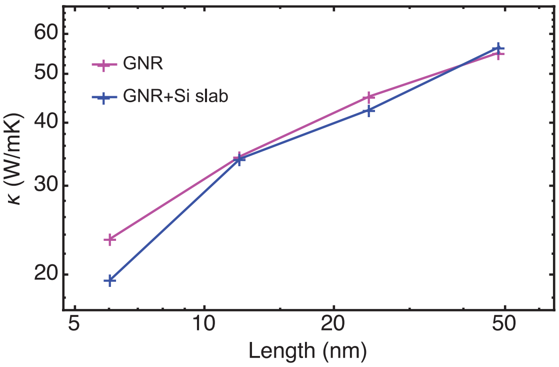

Figure S10: Length dependent κ_p of a planar GNR on top of a Si slab without constraint and the associated isolated GNR.

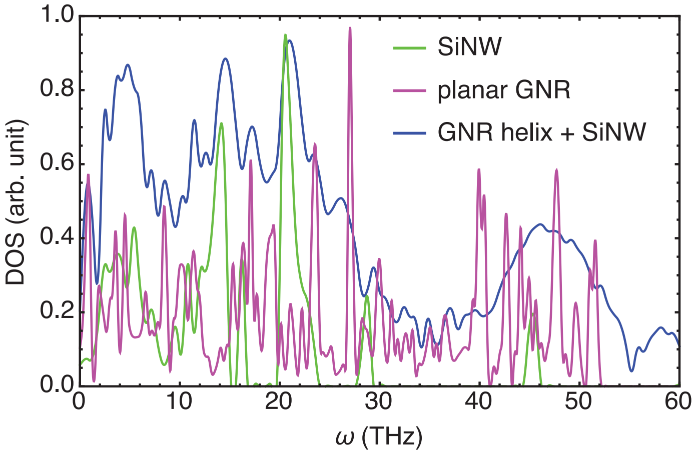

Figure S11: Phonon density of states of the isolated SiNW, planar GNR and combined GNR helix-SiNW system.

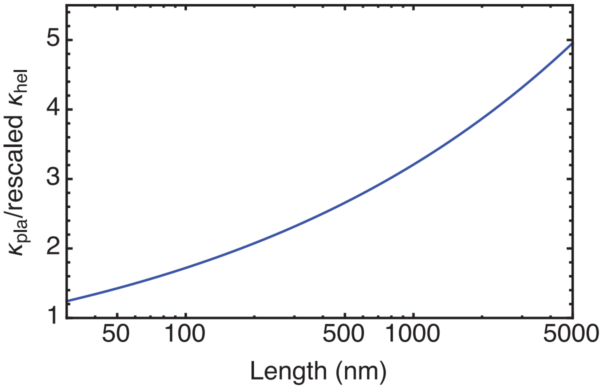

Figure S12: Length dependent reduction of rescaled κ_p in GNR helixes compared to that of planar GNRs.

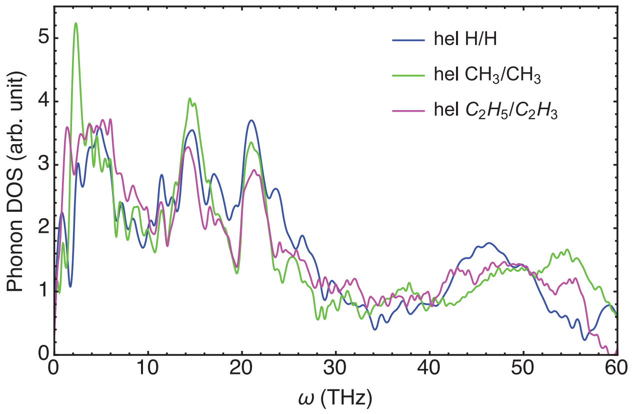

Figure S13: Phonon density of states of helix-SiNW systems with side chains introducing slight effects to κ_p.

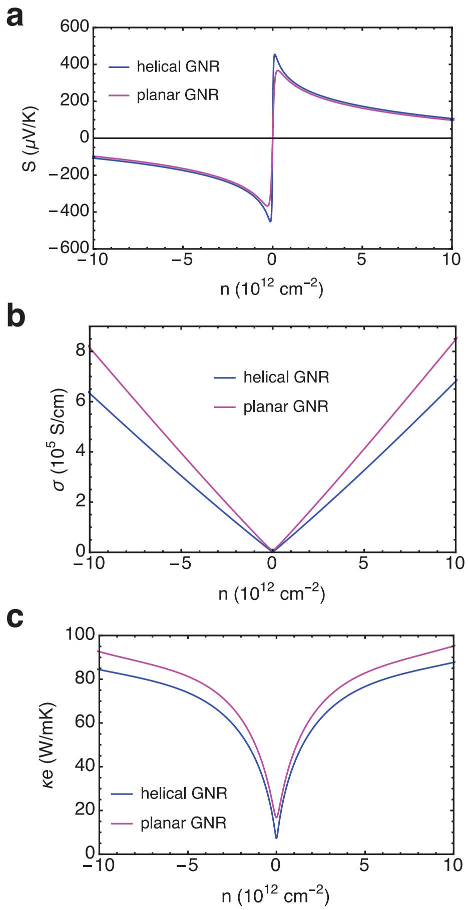

Figure S14: (a) Seebeck coefficient (S), (b) electrical conductivity (σ), and (c) electron contribution to thermal conductivity (κ_e) with respect to carrier concentration (n) of the planar and helical GNRs.

References

(1) Kresse, G.; Furthmüller, J. *Phys. Rev. B* **1996**, *54*, 11169.

(2) Blöchl, P. E. *Phys. Rev. B* **1994**, *50*, 17953.

(3) Perdew, J. P.; Burke, K.; Ernzerhof, M. *Phys. Rev. Lett.* **1996**, *77*, 3865.

(4) Monkhorst, H. J.; Pack, J. D. *Phys. Rev. B* **1976**, *13*, 5188.

(5) Kim, J. Y.; Grossman, J. C. *Nano Lett.* **2015**, *15*, 2830–2835.

(6) Perebeinos, V.; Avouris, P. *Phys. Rev. B* **2010**, *81*, 195442.

(7) Madsen, G. K.; Singh, D. J. *Comput. Phys. Commun.* **2006**, *175*, 67–71.

(8) Hwang, E.; Sarma, S. D. *Phys. Rev. B* **2008**, *77*, 115449.

(9) Sugihara, K. *Phys. Rev. B* **1983**, *28*, 2157.

(10) Fang, T.; Konar, A.; Xing, H.; Jena, D. *Phys. Rev. B* **2008**, *78*, 205403.

(11) Pennington, G.; Goldsman, N.; Akturk, A.; Wickenden, A. E. *Appl. Phys. Lett.* **2007**, *90*, –.

(12) Principi, A.; Vignale, G. *arXiv preprint arXiv:1406.2940* **2014**,

(13) Plimpton, S. *J. Comput. Phys.* **1995**, *117*, 1–19.

(14) Weitz, D.; Pine, D.; Pusey, P.; Tough, R. *Phys. Rev. Lett.* **1989**, *63*, 1747.

(15) Viscardy, S.; Servantie, J.; Gaspard, P. *J. Chem. Phys.* **2007**, *126*, 184513.

(16) Brenner, D. W.; Shenderova, O. A.; Harrison, J. A.; Stuart, S. J.; Ni, B.; Sinnott, S. B. *J. Phys. Condens. Matter* **2002**, *14*, 783.

(17) Stuart, S. J.; Tutein, A. B.; Harrison, J. A. *J. Chem. Phys.* **2000**, *112*, 6472–6486.

(18) Müller-Plathe, F. *J. Chem. Phys.* **1997**, *106*, 6082–6085.

(19) Zhang, M.; Lussetti, E.; de Souza, L. E.; Müller-Plathe, F. *J. Phys. Chem. B* **2005**, *109*, 15060–15067.

(20) Shiomi, J.; Maruyama, S. *Jpn. J. Appl. Phys.* **2008**, *47*, 2005.

(21) Yang, N.; Zhang, G.; Li, B. *Nano Today* **2010**, *5*, 85–90.

(22) Maruyama, S. *Phys. B: Condens. Matter* **2002**, *323*, 193–195.

(23) Li, B.; Wang, J. *Phys. Rev. Lett.* **2003**, *91*, 044301.

(24) Narayan, O.; Ramaswamy, S. *Phys. Rev. Lett.* **2002**, *89*, 200601_1–200601_4.

(25) Mingo, N.; Broido, D. *Nano Lett.* **2005**, *5*, 1221–1225.

(26) Nika, D. L.; Askerov, A. S.; Balandin, A. A. *Nano Lett.* **2012**, *12*, 3238–3244.

(27) Livi, R.; Lepri, S. *Nature* **2003**, *421*, 327–327.

(28) Chang, C.-W.; Okawa, D.; Garcia, H.; Majumdar, A.; Zettl, A. *Phys. Rev. Lett.* **2008**, *101*, 075903.

(29) Bae, M.-H.; Li, Z.; Aksamija, Z.; Martin, P. N.; Xiong, F.; Ong, Z.-Y.; Knezevic, I.; Pop, E. *Nature Commun.* **2013**, *4*, 1734.

(30) Kong, L. T. *Comput. Phys. Commun.* **2011**, *182*, 2201–2207.
